# Evaluating a Technology-Mediated HPV Vaccination Awareness Intervention: A Controlled, Quasi-Experimental, Mixed Methods Study

**DOI:** 10.3390/vaccines8040749

**Published:** 2020-12-10

**Authors:** Heather M. Brandt, Beth Sundstrom, Courtney M. Monroe, Gabrielle Turner-McGrievy, Chelsea Larsen, Melissa Stansbury, Karen Magradey, Andrea Gibson, Delia Smith West

**Affiliations:** 1 Department of Health Promotion, Education, and Behavior, Arnold School of Public Health, University of South Carolina, Columbia, SC 29208, USA; cmmonroe@mailbox.sc.edu (C.M.M.); mcgrievy@mailbox.sc.edu (G.T.-M.); 2 Department of Communication, College of Charleston, Charleston, SC 29424, USA; sundstrombl@cofc.edu; 3 Department of Exercise Science, Arnold School of Public Health, University of South Carolina, Columbia, SC 29208, USA; calarsen@email.sc.edu (C.L.); stansbm@email.sc.edu (M.S.); magradey@mailbox.sc.edu (K.M.); westds@mailbox.sc.edu (D.S.W.); 4 South Carolina Office of Rural Health, Lexington, SC 29072, USA; gibson@scorh.net

**Keywords:** human papillomavirus vaccination, cancer prevention, college students, social media, behavior change, health promotion intervention

## Abstract

College-aged women and men are an important catch-up population for human papillomavirus (HPV) vaccination interventions. Limited research has explored technology-mediated HPV vaccination awareness interventions aimed at college students. The purpose was to evaluate a novel, technology-mediated, social media-based intervention to promote HPV vaccination among college students. A controlled, quasi-experimental, mixed methods study examined the feasibility of a technology-based intervention among two undergraduate classes (*n* = 58) at a public university in the southeastern United States of America. Classes were randomized to receive one of two cancer prevention programs (i.e., HPV vaccination (intervention) or healthy weight (control)). Both programs contained eight technology-mediated sessions, including weekly emails and private Facebook group posts. Participants completed pre-/post-test surveys and submitted weekly qualitative reflections. Data were analyzed using descriptive statistics and thematic review for qualitative data. Knowledge improved among participants in the HPV vaccination intervention relative to those in the control condition. Participants (97%) interacted on Facebook by “liking” a post or comment or posting a comment. Participants demonstrated robust engagement and high treatment satisfaction. Results suggests that social media is an effective platform to reach college students with health promotion interventions and increase HPV vaccination awareness in this important catch-up population.

## 1. Introduction

Human papillomavirus (HPV) is pervasive and almost all men and women will contract it at some point in their lifetime [1]. HPV is the most common sexually transmitted infection (STI) in the United States (U.S.), and college students are at high risk for STIs. Young people, aged 15–24, account for almost half (49%) of new HPV infections in the U.S. [2]. In most cases, HPV infections clear without treatment; however, the virus can lead to genital warts and cancers in men and women, including cancers of the cervix, vulva, vagina, penis, and anus, and oropharyngeal cancer [3]. In 2015, there were 43,371 new cases of HPV-associated cancer in the U.S., with oropharyngeal cancer, which is more than twice as common in men, surpassing cervical cancer as the most common HPV-related cancer [4].

HPV vaccinations have been licensed by the Food and Drug Administration (FDA) since 2006 in the U.S. The currently available vaccine in the U.S., Gardasil^®^9, prevents up to 90% of HPV-related cancers [5]. HPV vaccines are effective and have significantly reduced rates of genital warts, HPV infections, and cervical lesions among women [6]. A recent surveillance study found evidence of HPV vaccine effectiveness and herd immunity among young women [7]. The Advisory Committee on Immunization Practices (ACIP) recommends a two-dose HPV vaccination series for both boys and girls between 11 and 12 years of age; however, in the U.S., uptake of the HPV vaccine is slow. From 2016 to 2017, HPV vaccination initiation among adolescents aged 13–17 increased from 60.4% to 65.5%, yet only 48.6% of adolescents were up-to-date with the HPV vaccine series in 2018 [8]. For adolescents and young adults who missed HPV vaccination at earlier ages (ages 15–26), the ACIP recommends a three-dose HPV vaccination series for women aged 15 to 26, men up to 21 years, and men ages 22–26 who have sex with men. HPV vaccination rates are particularly low in South Carolina, with only 42.7% of adolescents receiving the complete vaccine series (compared with 48.6% in the U.S.) [8]. Furthermore, South Carolina lags behind the U.S. among those receiving at least one dose of the HPV vaccine series (59.6% vs. 65.5%, respectively) [8]. Slow uptake of HPV vaccination results in a large population of college-aged men and women who are partially vaccinated or unvaccinated; this population constitutes what is commonly referred to as a “catch up” population (also called late vaccination). College-age women and men are at increased risk of acquiring HPV and are considered an important catch-up population for HPV vaccination [9,10].

Transitions from high school to young adulthood have been identified as a critical period to institute health-promoting behaviors [11]. College students are a critical population to target for HPV vaccine awareness and completion as these young adults begin to assume increased responsibility and independence surrounding healthcare decisions. Initial efforts to increase knowledge and awareness about HPV and appropriate vaccination practices have indicated promise, but point to the need for additional efforts to promote broader intentions to change and greater vaccine uptake [12,13,14,15,16,17].

Technology provides an attractive platform for HPV vaccination awareness interventions targeting college students because it is ubiquitous in their everyday lives. Nearly 100% of undergraduate college students use the internet [18]. Since 2013, internet use among young adults ages 18–29 years has remained at 97% or higher [19]. Furthermore, approximately 94% own a smartphone, 88% use social media [20], and 80% report using Facebook [21]. Behavioral interventions increasingly employ social media to improve awareness and vaccination coverage [22]. Recent studies have investigated HPV vaccination communication online through social platforms, including YouTube [23], Twitter [24], and Facebook [25]. Online messages about HPV vaccination include sensational and negative posts that may perpetuate misleading coverage and lead to insufficient information [23,25]. Therefore, effective online communication about HPV vaccination is needed to counter the dissemination of manipulative and false messages.

Interventions to increase HPV vaccination have historically targeted parents and health care providers [26,27,28]. However, a growing number of HPV vaccination interventions target young women and college students to increase vaccination rates among vaccine-eligible and catch-up populations [29,30,31,32,33,34,35]. Only a small number of HPV vaccination awareness interventions have employed social media, despite the dynamic potential and widespread reach of this platform [36,37,38,39,40]. A recent study found that a Facebook-assisted cervical cancer prevention discussion was more effective than an in-person discussion [41]. There is a clear opportunity for the development and evaluation of technology-based HPV vaccination interventions targeting college students.

The purpose of this study was to examine a novel, technology-mediated HPV vaccination awareness intervention for college students. Targeting college students during this transitional and formative period offers an opportunity to increase awareness and promote cancer prevention. The goal of this intervention was to encourage those in the catch-up population who had not yet completed vaccination to obtain the full HPV vaccine series.

## 2. Materials and Methods

### 2.1. Study Design and Procedures

A controlled, quasi-experimental, mixed methods study examined the feasibility of a technology-based intervention among two undergraduate classes at a public university in the southeast region of the U.S. This study included the implementation and evaluation of two health promotion and cancer prevention programs relevant to college students. Classes were randomized by coin flip to receive either an HPV vaccination awareness intervention or a behavioral weight gain prevention intervention (Healthy Weight; control). Each group served as the control for the other group, allowing for simultaneous intervention comparisons. The intervention was adapted from a successful theory-based health communication campaign, It’s My Time [42]. The study design and results from the Healthy Weight intervention arm have been published previously [43].

Both interventions included 8 technology-mediated health promotion sessions over 9 weeks, which included weekly emails, as well as private Facebook group posts. Study personnel posted to the private Facebook message board at least 5 times each week. Posts were scheduled to allow automated distribution through a social media management tool (Hootsuite Media Inc., Vancouver, BC, Canada) to ensure consistent delivery of content. Each group received the same schedule of email newsletters disseminated automatically using MailChimp (Rocket Science Group, LLC, Atlanta, GA, USA), which offered prescheduled distribution and tracked newsletter open rate. Participants completed a weekly open-ended reflection about the intervention emails and Facebook posts, which encouraged students to describe what factors influenced their personal engagement with the intervention. Posttreatment questionnaire data were obtained 9 weeks after intervention initiation. Detailed descriptions of the HPV and control groups are provided below.

This study was approved by the Institutional Review Boards at the College of Charleston and the University of South Carolina.

### 2.2. Participant Recruitment and Eligibility

Undergraduate students enrolled in an advanced health communication course were recruited via email. Students elected to voluntarily participate in the study or complete an alternative assignment for course credit. Students were not graded on engagement with the intervention. A description of the study and criteria for eligibility were provided. To be eligible, students were required to be registered in the course, have internet access on a computer or mobile device, and be willing to establish or use their current email address and Facebook account. Informed consent was provided via secure website by all students prior to participation.

All otherwise eligible college students enrolled in the two designated classes included in the study were eligible to participate, including those below the age of 18 years, so as to minimize any stigma or segregation of those college students who might not yet have turned 18 years of age. A waiver of parental permission for participation was requested and approved for those individuals below the age of 18 based on the minimal risk to the subjects associated with participation in the study and the lack of any adverse effects on the rights or welfare of the subjects, coupled with the difficulty in practically carrying out the project without this waiver. Because the selected courses were upperclass courses designed for college students further along in their studies, it was anticipated that there would not be any children below the age of 18. All participants were asked to sign (electronically) an informed consent form that was approved by the College of Charleston Institutional Review Board.

### 2.3. HPV Vaccination Awareness Intervention

The HPV vaccination intervention was based on the health belief model [44,45], the transtheoretical model [46], and adapted content from the successful theory-based health communication campaign, It’s My Time [42]. It’s My Time was developed through formative audience research and an academic-community collaboration to implement and evaluate a peer-based intervention. Formative research showed that preventing cancer is not a motivating message (or perceived benefit) to young adults. Therefore, the main message of It’s My Time encouraged participants to consider receiving the HPV vaccination so that they have time to pursue their dreams. Messages reinforced the concept of college students embracing their own time and time for themselves rather than emphasizing cancer risk-based messaging. A secondary message reminded students that it is not too late to receive the HPV vaccination or take control of their health [42].

These messages and health education information were incorporated into content that was delivered by weekly electronic newsletters and interactive Facebook posts in the current study. College students report limited knowledge of HPV and HPV-related cancer, coupled with high levels of concern about STIs [30,33,42,47]. Therefore, a key strategy of the intervention was to increase the perceived severity (i.e., the negative consequences) of contracting HPV and increase perceived susceptibility of acquiring HPV [26,31,34,42]. Campaign messages emphasized college students pursuing their interests and dreams with the time they gained from preventing HPV and HPV-related cancer. This approach aligns with current research that successful narrative messages depend on the perceived relevance of the storyline to the audience [31,48].

Lesson content focused on themes relevant to college students contemplating HPV vaccination (see Table 1). Messages were tailored to the campus and local area, with resources for how and where to obtain the HPV vaccination at low or no cost, including the university’s Student Health Services. Study investigators moderated the Facebook page, which included providing answers to questions and stimulating interaction, as well as sharing timely news articles and studies related to HPV vaccination. The posts were designed to cultivate a social climate of support for considering initiation of the vaccination series (or completion of the series for those who had partial completion of the sequence). Polls provided social norming to stimulate vaccination-related discussion. Intervention activities (e.g., reading the newsletters and interacting on Facebook) were intended to require approximately 30 total minutes per week. The program spanned a 9-week period during the spring semester, with a hiatus during spring break week.

### 2.4. Control Intervention

Similar intervention components were used for the control intervention. The control group received a Healthy Weight intervention, which included 8 electronic newsletters and a minimum of 5 postings per week to a separate, private Facebook group moderated by study staff to increase knowledge and support of healthy eating, activity, and weight control practices. Intervention content was derived from social cognitive theory [49] and established behavioral weight control programs, such as the Diabetes Prevention Program Lifestyle Intervention [50]. Emphasis was placed on key behavioral strategies for healthy eating and physical activity (e.g., goal-setting, self-monitoring, overcoming barriers, relapse prevention, and social support). Students were encouraged to take 10,000 steps/day and were provided with electronic tools for objectively monitoring their physical activity and weight (Fitbit Zip and Aria Wi-Fi-enabled scale, respectively; Fitbit Inc., Boston, MA, USA). The HPV and control interventions were matched on duration, structure, and number of planned contacts. However, the control intervention did not receive content related to HPV awareness or vaccination.

### 2.5. Measures

Participants completed assessments at baseline and post-intervention at 9 weeks. Questionnaires were administered online for all self-reported measures (Qualtrics, Provo, UT, USA).

#### 2.5.1. Sociodemographic Characteristics

Demographic characteristics were self-reported at baseline and included age, sex, race, and academic year. Sociodemographic characteristics included health insurance status, age at sexual debut, number of sex partners, gender of sex partners, relationship status, and contraceptive method.

#### 2.5.2. HPV Vaccination Knowledge, Attitudes, Beliefs and Behaviors

The current study adapted validated measures from Patel and colleagues [51], the 2013 Health Information National Trends Survey (HINTS), and the 2013 Behavioral Risk Factor Surveillance System (BRFSS). Survey items measured knowledge and awareness of HPV and HPV vaccination (e.g., “Some types of HPV can cause genital warts”; “People who have been infected with HPV might not have symptoms”), factors influencing the decision to be vaccinated (e.g., concerns about getting HPV, recommendation by a healthcare provider), and intention to seek HPV vaccination. In addition, students were queried about how many vaccines in the series they had received to date and, for those who had not yet completed the series, their intentions to seek out the vaccine.

#### 2.5.3. Intervention Engagement

HPV intervention engagement was assessed objectively on web-based platforms (MailChimp and Facebook). Engagement with HPV newsletters was defined as the number of students who opened the newsletter each week, which was obtained from MailChimp metrics. Engagement with the HPV Facebook group was measured by the number of interactions, including post likes, comments, and original posts. Interactions were aggregated for the week and tallied by research staff; the average number of interactions per week was determined.

#### 2.5.4. Treatment Satisfaction

At the conclusion of the intervention, students reported how satisfied they were with the HPV program by rating its overall usefulness and likelihood of recommending it to a friend or relative. Level of satisfaction with specific intervention components (e.g., number of sessions, frequency, and length of Facebook group postings) was also reported. All items were rated on an agreement-oriented 7-point Likert scale anchored with strongly agree and strongly disagree.

#### 2.5.5. Weekly Reflections

Participants received a weekly web-based survey inviting them to write a reflection critiquing the efficacy of each week’s materials and messages, including recommendations for improvement. Students were prompted to reflect on “how interesting”, “how useful”, and “how engaging” the newsletter and Facebook posts were each week. They were asked to provide specific examples and to incorporate concepts and theories from assigned readings and course discussions into their reflections.

### 2.6. Statistical Analysis

Descriptive statistics were used for all baseline and engagement measures. Analysis of treatment satisfaction aggregated agree, somewhat agree, and strongly agree responses, and combined disagree, somewhat disagree, and strongly disagree, and descriptive data are provided. One sample test of proportions was used to determine whether there was a significant difference in knowledge from pre-post for each group, and two sample test of proportions was used to determine whether there was a significant difference between the change in knowledge from pre-post between the two groups. A *p* value of less than 0.05 was used to determine statistical significance. All statistical analyses were performed using SPSS version 22.0 (IBM Corp., Armonk, NY, USA).

Qualitative data analysis of the open-ended weekly reflections was conducted using a constant comparative method [52], employing analytic tools recommended by Corbin and Strauss (2008) to ensure quality data coding. First, one author coded reflections line-by-line, which allowed new concepts to emerge. Then, a codebook was developed based on emergent concepts. Next, axial coding identified patterns and cross-cutting concepts. Researchers met frequently throughout the implementation and evaluation of the intervention and reached unanimous consensus on patterns, themes, and conclusions emerging from the data.

## 3. Results

### 3.1. Sample Characteristics

We utilized a convenience sampling approach. All registered students in two sections of the advanced health communication class were eligible for and chose to enroll in the study. Each section had 29 students. Participants (*n* = 58) included juniors and seniors with a median age of 21.6 ± 2.2 years. Most participants self-identified as female (81%) and white (90%). Most participants reported health insurance coverage on their parent’s plan (93%). Almost all participants reported initiating sexual activity (97%) and half of participants (50%) reported currently being in a monogamous partnership. Most participants reported using the oral contraceptive pill (62%) and/or the condom (52%) as a method of contraception. There were no significant differences in the baseline characteristics of students in the two groups. Retention rates at posttest were high, with no significant differences between conditions (see Table 2).

### 3.2. HPV Vaccination Knowledge, Attitudes, and Behaviors

At baseline, the majority of participants in the HPV vaccination intervention group (*n* = 19; 66%) reported having received all three shots of the HPV vaccine series (see Table 3). At posttest, two additional participants reported receiving all three shots of the HPV vaccine and an additional three participants indicated that they planned to get vaccinated in the next 6 months. As a result, at the conclusion of the intervention, 24 participants (83%) indicated that they had completed the HPV vaccination or had a plan to receive the HPV vaccination in the next six months. Differences in HPV vaccination between the intervention and control group were not significant, nor were there significant differences in the change between groups at posttest (see Table 3).

At baseline, almost all participants knew that HPV can be spread through sexual intercourse (97%). Fewer participants were aware that HPV can be spread through contact other than sexual intercourse (64%). Approximately 91% of participants correctly responded that people who have been infected with HPV might not have symptoms. While participants understood the link between HPV and cervical cancer (93%), fewer participants knew that some types of HPV can cause anal cancer (53%), oral cancer (57%), or genital warts (72%).

At posttest, knowledge of HPV and HPV vaccination improved among participants in the HPV vaccination awareness intervention relative to those in the control condition (Table 4). Almost all intervention participants reported that HPV can be spread through contact other than sexual intercourse (93%). Participants understood that some types of HPV can cause oral cancer (100%), anal cancer (93%), and genital warts (100%). These improvements in knowledge were statistically significant compared to the control group (see Table 4).

At pretest, approximately 35% of all participants reported that not being currently sexually active influenced their intention to receive the HPV vaccination. At posttest, only 7% of the HPV vaccination intervention group felt the same way, and the difference between the intervention and control group from pretest to posttest was significant (*p* = 0.03).

### 3.3. Participants Reported Improvements in HPV Vaccination Knowledge, Attitudes, and Behaviors

Participants’ open-ended weekly reflections demonstrated an increase in knowledge about HPV vaccination (see Table 5). According to one participant, “since the beginning of this intervention, my knowledge of HPV drastically increased.” Participants believed that the intervention addressed gaps in knowledge related to men’s susceptibility to HPV and the link between HPV and oral cancer. Many students also reported that prior to the intervention they did not realize Student Health Services provided the vaccine on campus. In addition to increasing knowledge, students reported that the intervention changed their beliefs about HPV vaccination. Participants also described how increased knowledge led to improved confidence and inspired conversations about HPV vaccination with family and friends. According to one participant, “I now feel extremely confident in discussing all aspects of HPV, including the importance of vaccination.”

### 3.4. Intervention Engagement and Treatment Satisfaction

**Figure 1:**
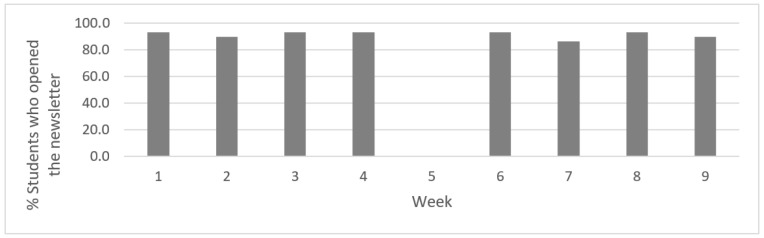
Participant engagement with HPV vaccination awareness newsletters (*n* = 29). Note: No electronic newsletters were sent by the study investigators during spring break (Week 5).

**Figure 2:**
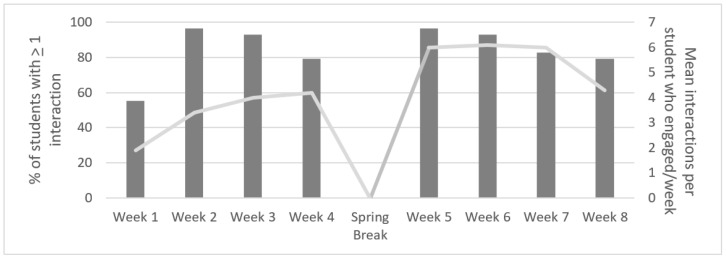
Participant engagement with HPV vaccination awareness intervention Facebook group. Note: No Facebook posts were made by the study investigators during spring break (Between Weeks 4 and 5).

Electronic newsletters were opened each week by the majority of participants in the HPV condition, with most participants (≥90%) opening each weekly newsletter (see Figure 1). The proportion of participants who had at least one interaction on the private Facebook page (i.e., liked or commented on a post shared by study investigators) was lowest in week 1 (55%), but increased in subsequent weeks, with the highest rates (97%) during weeks 2 and 5 (see Figure 2). Participants made a total of 906 comments and likes over the intervention period, resulting in an average of 4.5 ± 1.5 per person per week (not including spring break), with slight variation from week to week (see Figure 2). In total, participants averaged 31.2 ± 45.1 comments and likes over the course of the intervention. Providing health education through articles was well received by participants based on their feedback. In addition, posts seeking to normalize discussions of HPV prevention among peers by including polls, YouTube clips, and integrated other social media platforms received greater interaction than health education posts.

Participants in the HPV vaccine awareness condition rated the intervention positively. Overall, 89% of participants indicated the intervention was helpful, 78% said that they would recommend it to a friend, and 70% reported they enjoyed the program. Most participants (67%) reported that they were satisfied with the number of lessons, the number of Facebook postings (56%), the length of the Facebook postings (63%), and the extent of interaction with health educators on Facebook (81%).

### 3.5. Participants Reported High Intervention Engagement and Treatment Satisfaction

Based on the open-ended weekly reflections, Facebook emerged as an acceptable platform for the intervention (see Table 5). Students appreciated the accessibility, flexibility, and ease of use of the social media platform. According to one participant, “I liked the flexibility of using Facebook. It was easy to log in and jump into the discussion whenever my schedule allowed it.” Even students who did not previously have a Facebook account found the platform simple to use. Some participants believed that the intervention became repetitive over time, especially on Facebook. Other participants described “missing” posts on Facebook. According to one participant, “the Facebook content can be a little heavy; since Facebook does not have the most simple [*sic*] layout, it is hard to make a post really stand out.” Participants appreciated fun and interactive elements of the intervention, such as polls. Participants also commented that Facebook encouraged interaction. According to one participant, “I really enjoyed conversing and sharing opinions with peers via a social network. I felt more inclined to share more personal information because it was online versus a face-to-face conversation.”

We also monitored level of engagement with the Facebook HPV intervention content (see Table 6). These data were deidentified but monitored throughout the intervention period. One hundred percent of HPV intervention participants participated in the private HPV Facebook group as part of the HPV condition. Each week, an average of 23.9 participants (ranging from 16–28) actively engaged in the group. Active engagement was measured by how many individuals commented, liked any post, and liked comments that were made on posts. Level of engagement was stratified as the following: high (21 or more) medium (11–20), and low (0–10). On average, there were 8.2 comments (ranging from 0–24), 4.5 comment likes (ranging from 0–29), and 3.7 post likes (ranging from 0–13) per week. On the individual level, each participant commented in the group an average of 2.4 times per week (ranging from 0–9).

## 4. Discussion

To our knowledge, this is the first study to deliver an HPV vaccination awareness intervention through Facebook and electronic newsletters to college students. This technology-mediated, theory-based HPV vaccination awareness intervention was well accepted by participants. Findings suggest the approach was effective in improving knowledge about HPV and HPV vaccination. Participants reported that the intervention addressed gaps in knowledge related to men’s susceptibility to HPV and the link between HPV and oral cancer, which have been shown in previous studies to be important gaps limiting HPV vaccination [53]. Participants also described how increased knowledge led to improved confidence and inspired conversations about HPV vaccination with family and friends. At the start of the 9-week study, approximately half of participants had previously received the full HPV vaccination series (57%), which represents a considerably higher rate for full vaccination coverage than might have been expected based on estimates for adolescents in South Carolina (43%) [8]. This may reflect the high rates of insurance coverage in this sample since previous studies have indicated greater likelihood of completing the full HPV vaccination series among individuals with health insurance [54]. Despite the high levels of vaccination at baseline, almost half of the participants were inadequately protected from HPV and associated HPV-cancers, with rates far below the Healthy People 2020 goal of increasing HPV vaccination coverage to 80%.

At the conclusion of the HPV intervention, the number of participants initiating/completing the vaccination series increased, as did the number of young adults indicating they intended to get vaccinated in the next 6 months. The change in vaccination coverage was modest over this 9-week period and did not differ statistically from the control group in this small feasibility study. However, the demonstrated increase in knowledge about HPV and vaccination coupled with the suggestion of a shift in intensions to get vaccinated suggests that the intervention offers a promising approach to target the catch-up population and achieve the ambitious Healthy People 2020 80% full coverage goal.

Participants’ intervention engagement and treatment satisfaction suggest that this approach offers utility and scalability among college students. The electronic newsletter showed high penetration with approximately 90% of participants opening the email each week. Almost all participants interacted on Facebook by “liking” a post or comment or posting a comment. An average of more than four comments or likes were made per person each week throughout the intervention [55]. Participants reported that Facebook was easy to use, accessible, and flexible, which likely contributed to the success of the intervention. Students also described how an online social media platform encouraged interaction and personal sharing, which reinforced intervention messages.

### 4.1. Implications for Practice

Although HPV vaccination is effective, low coverage remains a challenge for public health professionals. College students represent an important catch-up population who are at increased risk of acquiring HPV. This technology-mediated intervention supports research that social media serves as an appropriate platform to improve awareness and vaccination coverage [22]. Intervention messages and materials countered the dissemination of manipulative and false messages online in real-time, providing a platform to address sensational and misleading coverage [23]. Findings support the promise of eHealth by filling a gap in the evaluation of theory-based programs measuring engagement in order to improve health behaviors and outcomes [56]. Specifically, this study evaluated objective measures and qualitative in-depth reflections of engagement to show that the intervention resonated with participants.

### 4.2. Study Limitations and Strengths

Findings should be understood within the context of the study’s limitations. The small sample size and homogeneity of the population limit the generalizability of the results. The relatively short intervention and lack of extended follow-up to confirm receipt of HPV vaccinations are limitations of this study. Additionally, the relatively high baseline rate of full vaccination left modest room to increase vaccination rates. Finally, there is a risk of contamination between conditions because students were registered in two sections of the same course, with the same professor, at the same university and both interventions were described in the informed consent document.

This pilot study offers a number of strengths that demonstrate potential for scalability and dissemination of this intervention approach. In one of the first HPV vaccination awareness interventions for college students delivered through electronic newsletters and Facebook, participants demonstrated robust engagement and high treatment satisfaction. Moreover, the study was randomized and controlled, with a high retention rate. Additional study strengths include the use of objective measures and qualitative open-ended assessment of intervention engagement. Finally, the automated delivery of electronic newsletters and Facebook posts represents an opportunity to replicate the intervention with limited time and opportunity cost.

## 5. Conclusions

A technology-mediated HPV vaccination awareness intervention increased college students’ knowledge of HPV and HPV vaccination. Objective measures and qualitative open-ended assessment of the intervention showed high levels of engagement with the electronic newsletters and Facebook group. The effectiveness of this pilot study suggests that social media is an appropriate platform to reach college students with health promotion interventions and increase HPV vaccination awareness in this important catch-up population.

## Figures and Tables

**Table 1:** Weekly lesson topics delivered through electronic newsletters and Facebook posts.

Session	Topics
1	What is HPV?
2	Am I at risk for HPV?
3	How do I prevent HPV?
4	How do I know if I have HPV?
5	What are the risks and benefits of HPV vaccination?
6	Should I get the HPV vaccine?
7	Where do I go to get the HPV vaccine?
8	Why do I need 3 doses?

**Table 2:** Baseline characteristics and retention rates.

Measure	Total (*n* = 58)	HPV (*n* = 29)	Control (*n* = 29)
Age (SD), years	21.6 (2.2)	21.1 (0.8)	22.1 (2.9)
Sex, *n* (%)			
Female	47 (81)	24 (85)	23 (79)
Male	11 (19)	5 (17)	6 (21)
Hispanic, *n* (%)			
Yes	5 (9)	3 (10)	2 (7)
No	53 (91)	26 (90)	27 (93)
Race, *n* (%)			
Caucasian	52 (90)	28 (97)	24 (83) *
African-American	3 (5)	1 (3)	2 (7)
Asian	2 (3)	0 (0)	2 (7)
American Indian/Native American	1 (2)	0 (0)	1 (3)
Academic year, *n* (%)			
2nd year	1 (2)	0 (0)	1 (3)
3rd year	17 (29)	13 (45)	4 (14)
4th year	33 (57)	14 (48)	19 (66)
5th year	7 (12)	2 (7)	5 (17)
Health Insurance Status, *n* (%)			
Parent’s Plan	54 (93)	28 (97)	26 (90)
None	3 (5)	1 (3)	2 (7)
Another plan	1 (2)	0 (0.0)	1 (3)
Age at first sexual intercourse (SD), years	16.9 (1.6)	17.1 (1.8)	16.7 (1.3)
Number of sex partners (SD)			
Past month	0.9 (0.6)	10 (0.6)	0.9 (0.6)
Past 6 months	1.9 (2.1)	1.7 (1.2)	2.1 (2.7)
Lifetime	7.0 (5.7)	6.6 (5.0)	7.5 (6.4)
Current monogamous partnership			
Yes	28 (50)	14 (48)	14 (52)
No	26 (46)	14 (48)	12 (44)
Don’t Know	2 (4)	1 (3)	1 (4)
Retention rates	56 (97%)	29 (100%)	27 (93%)

* *p* value < 0.05.

**Table 3:** HPV vaccination status and intentions.

	HPV (*n* = 29)		Control (*n* = 29)	
	Pre	Post	Pre	Post
Received full vaccination series (%)	19 (66)	21 (78)	14 (48)	15 (52)
Initiated partial vaccination series (%)	2 (7)	0 (0)	3 (10)	4 (14)
Never vaccinated (%)	7 (24)	3 (11)	9 (31)	8 (28)
Intend to get vaccinated within 6 months (%)	1 (3)	3 (11)	3 (10)	2 (7)

Fisher’s exact test was used to assess whether there were significant differences between and within groups at pre and post. No significant differences were found.

**Table 4:** HPV and HPV vaccination knowledge (%).

	HPV		Control		
	Pre (*n* = 28)	Post (*n* = 27)	Pre (*n* = 29)	Post (*n* = 29)	Difference in Change between Groups at Posttest (*p*-Value)
Some types of HPV can cause oral cancer. Correct answer: True	65.5	100 *	48.3	69.0	0.007
Some types of HPV can cause anal cancer. Correct answer: True	58.6	92.6 *	48.3	65.5	0.04
Some types of HPV can cause genital warts. Correct answer: True	78.6	100 *	69.0	82.8	0.024
HPV can be spread through contact other than sexual intercourse. Correct answer: True	69.0	92.6 *	58.6	69.0	0.072
Condom use fully protects against the spread of HPV. Correct answer: False	69.0	92.6	75.9	82.8	0.42
HPV can cause an abnormal Pap (cervical cancer screening) test. Correct answer: True	82.8	96.3	82.8	86.2	0.186
People who have been infected with HPV might not have symptoms. Correct answer: True	86.2	100	96.6	96.6	0.33
HPV can be spread through sexual intercourse. Correct answer: True	100	100	93.1	100	1.00
Some types of HPV can cause cervical cancer. Correct answer: True	96.6	100	89.7	93.1	0.165
An HPV infection can be cured. Correct answer: False	27.6	37	65.5	41.4	0.049
Women who get the vaccine still need regular Pap (cervical cancer screening) tests. Correct answer: True	93.1	85.2	93.1	86.2	0.76

* *p* value < 0.05.

**Table 5:** Themes with illustrative quotes.

Themes	Illustrative Quotes
HPV vaccination knowledge, attitudes, and behaviors	“After reading all of the posts and doing some other research I now believe that the vaccine is something everyone should have. I mean there is really no reason not to get vaccinated.” “My viewpoint has definitely changed for the better. At the beginning of this study, I did not know too terribly much about HPV. At this point I know quite a bit and I know the facts concerning the matter. I’ve been able to relay this information to my friends and family and help them even take steps in the right direction to protect themselves.”
Intervention engagement and treatment satisfaction	“I believe the material presented in this week’s Facebook post were user friendly and easy to understand and read. I am not familiar with the use of Facebook but I found it pretty easy to use and follow along with.” “Many students even got to share personal stories of people who have had issues with HPV in the past. I believe this is important to share because if you personally know someone effected by HPV or even if it is just a friend of a friend, the situation or topic of HPV automatically becomes more personal and ‘hits home’ for a lot more people. I also enjoyed reading other people’s articles that they have found from other sources. The more information or reading on this topic helps for me to get a better idea of truly how serious HPV is.”

**Table 6:** Level of engagement with Facebook HPV intervention content.

Level of Engagement	Comments Number of Students	Post Likes Number of Students	Comment Likes Number of Students
High (21+)	11	1	4
Medium (11–20)	12	6	5
Low (0–10)	6	22	20
